# Direct Recovery of the Rare Earth Elements Using a Silk Displaying a Metal-Recognizing Peptide

**DOI:** 10.3390/molecules25030761

**Published:** 2020-02-10

**Authors:** Nobuhiro Ishida, Takaaki Hatanaka, Yoichi Hosokawa, Katsura Kojima, Tetsuya Iizuka, Hidetoshi Teramoto, Hideki Sezutsu, Tsunenori Kameda

**Affiliations:** 1 Strategic Research Division, TOYOTA Central R&D Labs, Inc., 41-1, Yokomichi, Nagakute, Aichi 480-1192, Japan; n-ishida@mosk.tytlabs.co.jp (N.I.); takaaki-h@mosk.tytlabs.co.jp (T.H.); e1305@mosk.tytlabs.co.jp (Y.H.); 2 Silk Materials Research Unit, Division of Biotechnology, Institute of Agrobiological Sciences, National Agriculture and Food Research Organization (NARO), 1-2, Owashi, Tsukuba, Ibaraki 305-8634, Japan; kojikei@affrc.go.jp (K.K.); teramoto@affrc.go.jp (H.T.); 3 Transgenic Silkworm Research Unit, Division of Biotechnology, Institute of Agrobiological Sciences, National Agriculture and Food Research Organization (NARO), 1-2, Owashi, Tsukuba, Ibaraki 305-8634, Japan; tiizuka@affrc.go.jp (T.I.); hsezutsu@affrc.go.jp (H.S.)

**Keywords:** rare earth elements, dysprosium, transgenic silkworm, rare earth recovery

## Abstract

Rare earth elements (RE) are indispensable metallic resources in the production of advanced materials; hence, a cost- and energy-effective recovery process is required to meet the rapidly increasing RE demand. Here, we propose an artificial RE recovery approach that uses a functional silk displaying a RE-recognizing peptide. Using the *piggyBac* system, we constructed a transgenic silkworm in which one or two copies of the gene coding for the RE-recognizing peptide (Lamp1) was fused with that of the fibroin L (FibL) protein. The purified FibL-Lamp1 fusion protein from the transgenic silkworm was able to recognize dysprosium (Dy^3+^), a RE, under physiological conditions. This method can also be used with silk from which sericin has been removed. Furthermore, the Dy-recovery ability of this silk was significantly improved by crushing the silk. Our simple approach is expected to facilitate the direct recovery of RE from an actual mixed solution of metal ions, such as seawater and industrial wastewater, under mild conditions without additional energy input.

## 1. Introduction

Rare earth elements (RE), which show special magnetic and optical properties owing to their distinctive electronic features, are indispensable metallic resources; they can be used in the production of various devices such as permanent magnets in hybrid and electric cars, mobile phones, light-emitting diode lamps, and motors of wind turbines. Given the increasing demand for RE metals, the main challenge is to meet this demand by increasing the supply [1,2]. China is the major supplier of RE metals; however, the mining process requires a more efficient separation and recovery process owing to the increasing risk of environmental pollution [3]. Several advanced techniques involving solvent extraction and ion chromatography have been reported in an attempt to make the process more environmentally and economically friendly [1,2]. Furthermore, lanthanides, which consist of 15 elements from lanthanum (La) to lutetium (Lu), show very similar characteristics; the challenge of selective separation between these lanthanide elements has also been highlighted [4]. In particular, the separation of dysprosium (Dy) and neodymium (Nd) found in the permanent magnets from scrap is a major problem. Permanent magnets containing Dy and Nd are used in motors of hybrid and electric cars, and therefore environmentally friendly processes of metal recovery and recycling are considered important to meet the ever-increasing demand. To overcome this issue, several advanced studies have been reported; in these reports, the extraction and separation of Ln from other Ln species have been achieved using novel organic compounds that exhibit ionic radii selectivity [1]. In addition, approaches using metal ligand have been reported for RE separation. For example, a metal complex of tripod nitroxide ligand [5], coordination polymers that form extended complexes with metal ions containing organic ligands [6], and a metal-organic framework (MOF) [7] have been successfully used to separate Nd and Dy based on size-sensitive dimerization. However, these approaches require environmentally unfavorable artificial treatments, such as low pH, high temperatures, and the use of organic solvents. Hence, novel RE recovery techniques that are energy- and cost-effective are required.

In our previous study, we proposed a novel recovery process using an RE recognition peptide inspired by biomineralization [8]. Considering the chemical equilibrium of Ln^3+^ in aqueous solution, we screened peptide binding to the Ln-hydroxide (hydro-oxide) using the phage display. Identified peptides with cyclic structures, Lamp1: lanthanide ion mineralization peptide 1 (SCLWGDVSELD-FLCS), recognize the lanthanide ions (Ln^3+^) and instantaneously convert them into the precipitates of lanthanide hydroxides (Ln(OH)_n_) without the use of organic solvents. The major driving force in the Ln^3+^ recognition of this peptide is the charge interactions from the side-chain carboxylic acid groups of the acidic amino acids (aspartic acid and glutamic acid). Simultaneously, precipitation occurs due to self-assembly, which is promoted by the hydrophobic nature of the formed Ln complex. The heavy Ln^3+^ hydroxides show preferential precipitation over the light Ln^3+^ species. This feature could be particularly useful in solving the challenging problems related to the selective separation of 15 lanthanides. By using sepharose resin conjugated with Lamp1, we demonstrated the potential of this approach to selectively separate Ln^3+^ from seawater without the use of organic solvents. In addition, the RE once adsorbed on the material can be recovered easily with a weakly acidic solution; moreover, this sepharose resin can be recycled multiple times. This simple process is, therefore, an environmentally friendly and low energy alternative for the recovery of RE elements.

In this work, we propose an environment friendly RE recovery approach using functional silk displaying a metal-recognizing peptide, Lamp1. Silk is a natural fiber produced by the silkworm which is a classic model organism for endocrinology. The silkworm has been reared by humans for thousands of years, and more than 1000 strains are currently maintained [9]. Silk is a fiber protein that can be mass-produced through sericulture techniques. Silk consists of hydrophilic protein (sericin), two kinds of hydrophobic proteins (fibroin heavy chain, FibH and fibroin light chain FibL), and glycoprotein (P25/fibrohexamerin) [10]. Silk, solubilized in aqueous media, can be processed into particles, gel, sponges, and filters. New silk materials have been designed for use in clothing, cosmetics, and medical applications [11,12,13,14]. Moreover, since the establishment of a genetic recombination system of *Bombyx mori* using *piggyBac*, which is the transposable element from the cabbage looper *Trichoplusia ni* TN-368 cells [15], many trial studies on silk fibers fused with proteins have been reported for functional materials [16,17,18,19]. As a representative attempt, the production of large quantities of fluorescent-colored silks by expressing green fluorescent protein (GFP) has already been established [16]. Another study has been reported, which attempts to produce more durable silk by fusing it with the protein of spider dragline silk [18]. On the basis of these advanced techniques, the present work shows the possibility of direct recovery of the RE elements using a silk fiber displaying a RE-recognizing peptide. We constructed a transgenic silkworm producing a FibL fused with Lamp1 and evaluated the direct recovery of the RE using these fusion proteins. We expect that our results should contribute to the possibility of using direct RE recovery under mild conditions without additional energy input.

## 2. Results and Discussion

### 2.1. Breeding of Transgenic Silkworms

**Figure 1:**
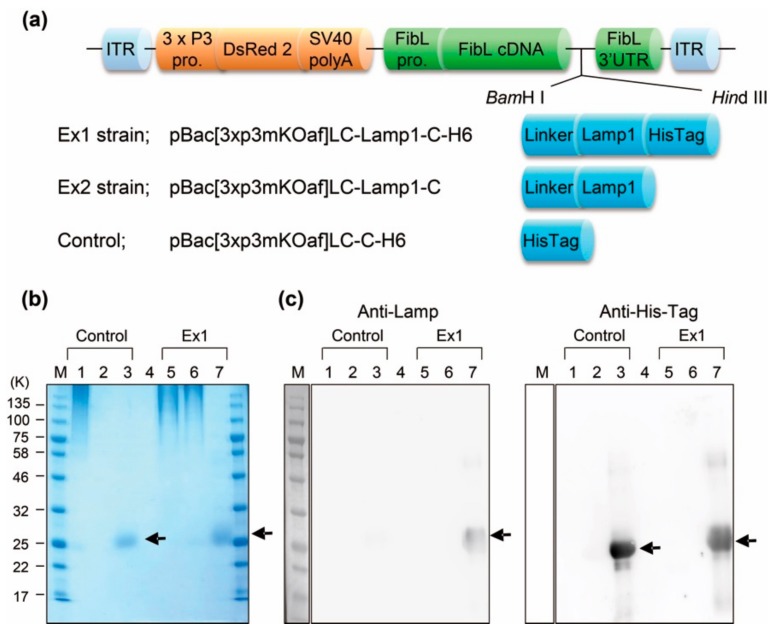
Breeding of the transgenic silkworm. (**a**) Construction of the expression vector. Each plasmid contains expression units for selection marker gene (*DsRed2*) and cDNA of fibroin L-chain (*FibL* cDNA) between the *piggyBac* inverted terminal repeat (ITR). Three gene fragments containing Lamp1 or His Tag were inserted into the 3’ ends of *FibL* cDNA using restriction site of *Bam*H I and *Hin*d III. 3xP3 pro., 3xP3 promoter; SV40 polyA, polyA signal of SV40; FibLpro., promoter of fibroin L-chain; and FibL 3’ UTR, 3’ untranslated region of fibroin L-chain. (**b**) SDS-PAGE analysis of purified FibL-Lamp1-His protein from Ex1 strain and the control strain. (**c**) Western blotting analysis using Lamp1, and His-tag antibody. Each arrow indicates the expected molecular weight of the target protein. Lane 1, FibL sample before purification; Lane 2, flow-through of FibL column; Lane 3, purified FibL; Lane 4, buffer; Lane 5, FibL-Lamp1 sample before purification; Lane 6, flow-through of FibL-Lamp1 column; and Lane 7, purified FibL-Lamp1.

In order to generate transgenic silkworms, we constructed an integration vector wherein the gene encoding FibL was fused with that of cyclic peptide, Lamp1. The reason for selecting FibL is that it is the most widely reported of the fibroin fusion proteins, which has the advantage that various genomic integration vectors have been developed and transgenic silkworms can be obtained with a high efficiency [20]. As elucidated in Section 2.3., in general, in the *piggyBac* system, the amount of the FibL fused to the foreign proteins is estimated to be about 10%, or less, of the total FibL, and the remaining 90%, or more, is occupied by native FibL. Therefore, a vector (FibL-Lamp1-His) that can introduce a histidine (His) tag at the N-terminus of Lamp1 was also constructed to separate and purify only the protein fused with Lamp1. Another vector that produces FibL fused with His tag only (without Lamp1) was also constructed as a control. Three kinds of *piggyBac* vectors, pBac[3 xp3mKOaf] LC-Lamp1-C-H6, pBac[3xp3mKOaf] LC-Lamp1-C, and pBac[3xp3mKOaf]LC-C-H6, were designed to express red fluorescent protein under the control of the 3xP3 promoter (Figure 1a). Using *DsRed2*, chromosomal integration of the *FibL-Lamp1* can be confirmed by the change of an eye color to red. The vectors were injected into the pre-blastoderm embryo, and the obtained larvae (G0) were bred for several months. The desired G1 generation was screened for *DsRed2* expression in the eyes of embryo; subsequently, the G2 generation (homothallic strain) was obtained by multiplying G1 generation. We named these constructed strains Ex1, Ex2, and control (Figure 1a). The final transgenic efficiency was approximately 25.8%, 17.5%, and 33.3%, respectively. This efficiency was comparable to previous studies using the *piggyBac* system [11]. As a result of genomic southern blot analysis with the Ex1 and Ex2 strains, the existence of one or two copies of the target *FibL-Lamp1* was confirmed in all 24 homothallic silkworms (Supplementary Figure S1). Each band of Southern blot was observed at the same positions in most individuals, suggesting that the *FibL-Lamp1* was inserted at the same position on the chromosome.

To investigate the expression of target FibL-Lamp1, Western blotting was performed and analyzed for the Ex1 and control strains. Solubilized silk protein was prepared by lithium bromide treatment and target FibL-Lamp1 protein was purified using His-tag. The SDS-PAGE analysis showed the separation of FibL and FibH, and a single fragment was observed in the protein sample after column purification (Figure 1b). Since the molecular weight of Lamp1 is 1.67 kDa, the total molecular weight of the fusion protein including the linker peptide is estimated to be about 27.23 kDa. Each single fragment detected by SDS-PAGE analysis indicates the expected molecular weight. In the Western blot analysis using two types of antibodies, an anti-Lamp and anti-His Tag, 27.23 kDa fragment was detected as the major protein, which is the expected molecular weights in the Ex1 strain (Figure 1c). In addition, in the control strain without Lamp1, no fragment was detected upon probing with the anti-Lamp.

### 2.2. RE Recognition with the Purified Fusion Protein

**Figure 2:**
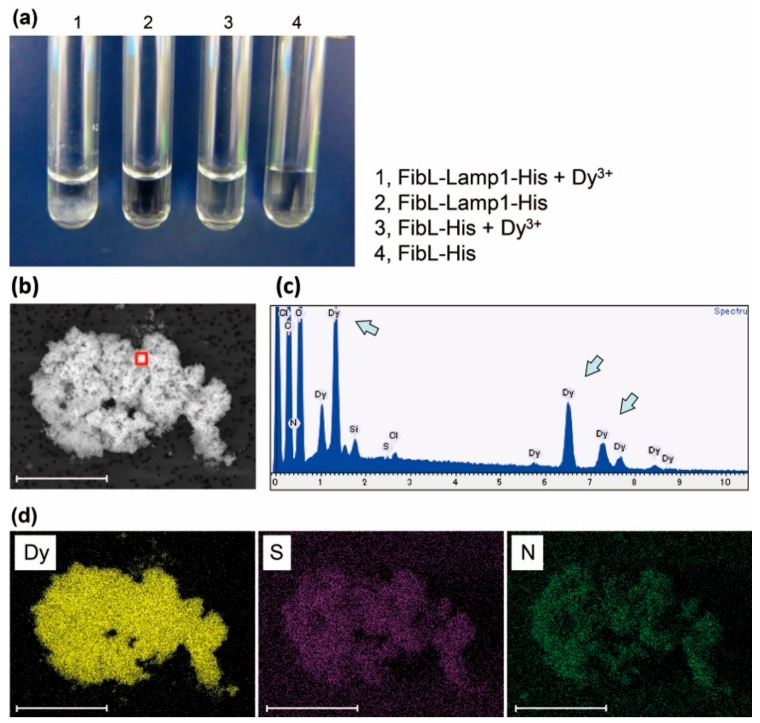
Dy^3+^ recognition with purified FibL-Lamp1-His protein. (**a**) Optical image of precipitation induced with protein adding. (**b**) SEM image of precipitation. Scale bar, 100 µm. (**c**) EDX spectrum. The red frame area in the SEM image of (b) was analyzed. Each arrow indicates the spectrum of Dy. (**d**) Elemental mapping of SEM image (Dy, dysprosium; N, nitrogen; and S, sulfur). Scale bar, 100 µm.

**Figure 3:**
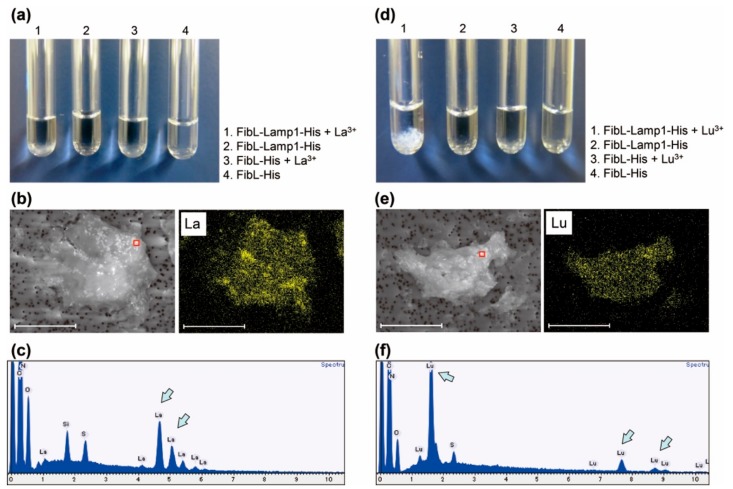
La^3+^ and Lu^3+^ recognition with the purified FibL-Lamp1-His protein. (**a**–**c**) Reaction with La^3+^, and (**d**–**f**) reaction with Lu^3+^. (**a**–**d**) Optical image of precipitation induced by the purified FibL-Lamp1-His protein. (**b**–**e**) SEM image (left) and elemental mapping image (right) of precipitation. Scale bars, 100 µm. (**c**–**f**) EDX spectrum. The red frame area in the SEM image was analyzed. Each arrow indicates the spectrum of La or Lu.

When 5 mM Dy^3+^ was mixed to the purified 50 µM FibL-Lamp1-His protein, the solution immediately became cloudy and a precipitate was observed in a few minutes at pH 6.0 (Figure 2a). In contrast, little or no precipitation was observed with the negative control without Lamp (FibL-His) under the same conditions. Scanning electron microscope and energy dispersive X-ray spectroscopy (SEM-EDX) analysis of the thoroughly washed precipitate showed a clear signal indicative of Dy (Figure 2b–d). We also investigated the recognition of other light and heavy RE, such as La^3+^ and Lu^3+^, and the similar precipitation was observed with the clear spectrum indicating the presence of either La or Lu (Figure 3). Visual observation of the results obtained for the three elements suggested that the large amount of precipitate was formed upon addition of Lu^3+^. This tendency of heavy REs to precipitate preferentially over light REs is in agreement with our previously reported studies using Lamp1 [8]. Separation of lanthanides is a major challenge because 15 lanthanide ions exhibit very similar characteristics [4]. Although Lamp1 has the properties of recognizing RE ions, separation of lanthanides is still challenging, and further changes of the peptide sequence for improving selectivity are required.

**Figure 4:**
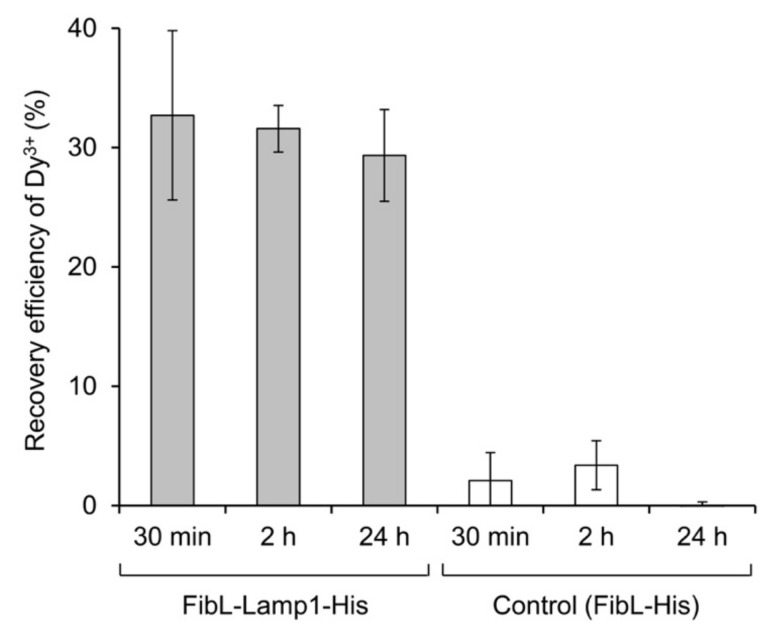
The recovery efficiency of Dy^3+^ with the purified protein. The supernatant solution after reaction (mentioned in Figure 2a) was collected, and the Dy^3+^ concentration in the solution was determined. Average and standard deviations from three independent experiments were represented.

We examined the recovery of Dy, which is an essential element for improving the performance of permanent magnets. To assess the recovery using FibL-Lamp1-His protein, the amount of Dy^3+^ remaining in the supernatant was quantified via ion-exchange chromatography (ICE). The lanthanide ion is characterized by the formation of a complex having an absorption wavelength at 520 nm when mixed with 4-(2-pyridylazo) resorcinol (PAR) [21]. Using this complex, the analytical method was established by ICE [22,23]. The recovery efficiency of Dy^3+^ adsorbed to purified FibL-Lamp up to 24 h was approximately 32.7% (Figure 4). Therefore, the Dy^3+^ adsorption capacity was found to be approximately 32.7 µM per micromolar of the purified protein. There was no significant difference at 30 min and 24 h after the initiation of the reaction in this condition.

### 2.3. Direct Recovery of RE using Silk Powder

**Figure 5:**
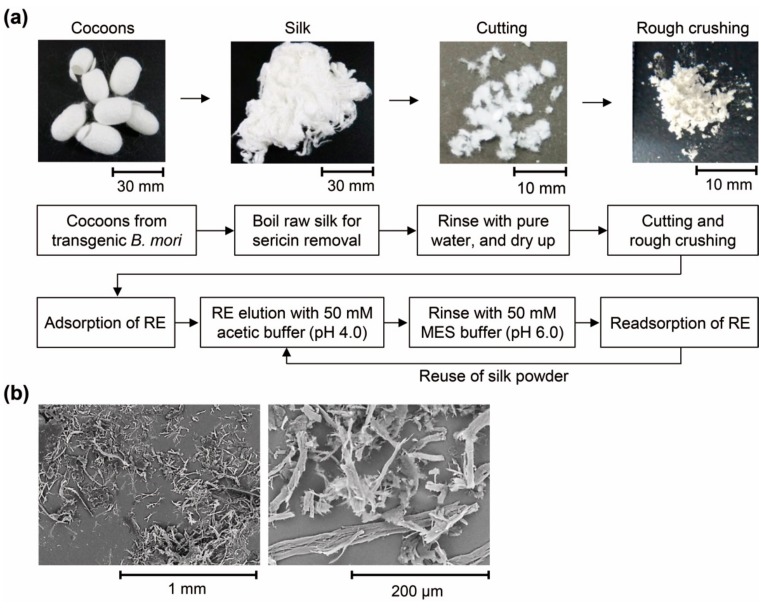
RE recovery process with silk powder: (**a**) Schematic diagram of the process for RE-recovery, and optical image of the prepared silk and (**b**) SEM image of roughly crushed silk.

The potential for direct RE recovery was examined using the silk producing Ex2 strain. The silk derived from the homothallic Ex2-2 strain, which produces the largest amount (20 g or more) of silk among the transgenic lines of Ex2, was used for the examination of RE recovery. In this analysis, silk produced by wild-type silkworms was used as a control. The RE recovery process was conducted as shown in Figure 5a. In order to make Lamp1 fuse with FibL function effectively, sericin on the surface of raw silk was removed through boiling. Two types of samples were prepared. The first was prepared by cutting approximately 5 to 10 mm with scissors and a second was prepared by rough crushing using a ball mill mixer. From the sample processed with a ball mill, a pulverized product having a length of approximately 200 to 500 µm was obtained (Figure 5b).

**Figure 6:**
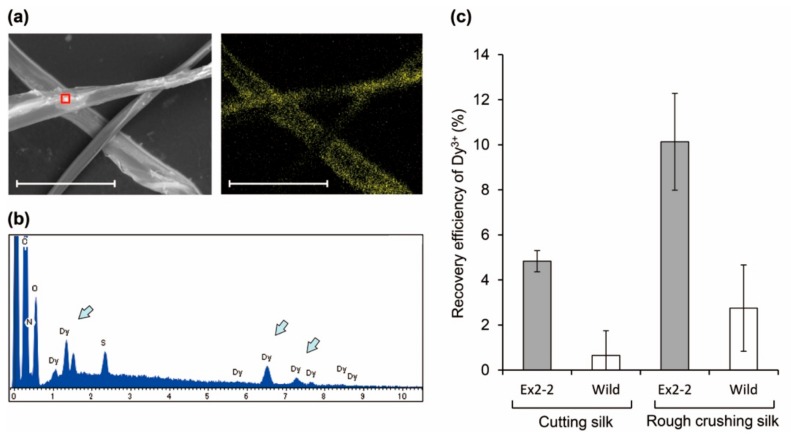
Dy^3+^ recognition with silk powder: (**a**) SEM (left) and elemental mapping (right) images of silk powder after Dy^3+^ absorption reaction. The yellow signal indicates a Dy. Scale bar, 80 µm, (**b**) EDX spectrum, each arrow indicates the Dy signal and (**c**) the recovery efficiency of Dy^3+^ of silk with harvested silk from Ex2-2. Averages and standard deviations for three independent experiments were represented.

**Figure 7:**
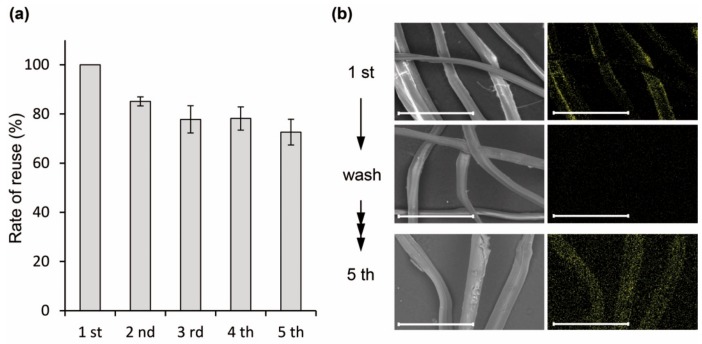
Reusability of silk powder for Dy^3+^ recovery. (**a**) The relative degree to the initial Dy^3+^ recovery efficiency is shown. Averages and standard deviations for three independent experiments were represented. (**b**) SEM-EDX analysis, the captured Dy (yellow signal) was eluted with acetic buffer (50 mM) at pH 4.0, and the silk powder was recycled. Scale bars: 100 µm.

After adding 5 mM Dy^3+^ to the 20 mg silk powder at pH 6.0, a clear signal indicative of Dy was detected by SEM-EDX, similar to the purified protein (Figure 6a,b). Interestingly, the cut silk showed approximately 5% of Dy^3+^ recovery, which was improved to 11% by rough crushing with a ball mill (Figure 6c). This value indicated that the Dy^3+^ adsorption capacity was approximately 27.5 µM per mg of the silk powder. The reason is that the ratio of Lamp1 presented on the fiber surface was improved by crushing. Although this efficiency was low as compared with the purified protein, the advantage is that the RE can be recovered directly with silk. Moreover, slight Dy^3+^ adsorption was also observed in the control, which was attributed to the presence of hydroxyl group on the fiber surface. To examine the reusability of the silk powder, once the Dy^3+^ was adsorbed to the silk surface it was washed off and reacted again with the new Dy^3+^. Previously, in our study using Lamp1 immobilized sepharose resin, we demonstrated that Dy^3+^ once adsorbed by Lamp1 can be released by washing with acetate buffer at pH 4.0 [8]. Following the same method, reusability of rough crushing powder was performed, showing the relative degree to the initial Dy^3+^ recovery efficiency. In the five rounds of recycling, the recovery efficiency of Dy^3+^ decreased by approximately 20% (Figure 7), potentially because of the loss of particles upon multiple centrifugation and washing steps. However, no significant reductions were observed thereafter, confirming the potential for repeated use.

Regarding the expression of peptide-fused silk in transgenic silkworms, several studies have been conducted, which includes the expression of antibacterial peptides, as well as cell growth peptides [17,24]. The successful use of Lamp1 peptide in this study promises the development of other metal recovery silk by using a variety of metal recognition peptides. However, further advancement is required in terms of the cost, energy, and practicability to make the efficient use of the proposed RE recovery process on a large scale. In order to further improve the performance of metal recovery silk, it is necessary to increase the concentration and availability of the peptide presented on the fiber surface by controlling the expression and molecular orientation of the fusion protein, respectively. The fibroin molecules consisted of FibH, FibL, and P25 protein in a molecular ratio of 6:6:l and the molecular weights of the each protein are around 400, 30, and 25 kDa, respectively [10]. In the *piggyBac* system, because the target *Lamp1* are integrated randomly on the chromosome regions separately from the native *FibL* gene, the content of the FibL fused to the foreign proteins is estimated to be about 10% or less in the total FibL, and the remaining 90% or more is occupied by the native FibL [25]. On this basis, the molecular ratio of FibL-Lamp1 in the total silk fiber is considered to be only about 4.61% or less. For the efficient production of transgenic fusion protein, an interesting approach utilizing *Nd-s^D^* mutant strain lacking part of the *FibL* gene in the chromosome has been proposed to increase the proportion of recombinant protein in silk. Using this strain for transgenic silkworm, all fibroin was replaced by fusion protein, indicating improved productivity of foreign proteins [25]. In another approach, genome editing technology, that enables gene integration to the target position in the chromosome has also been developed in *B. mori* [26,27,28]. Efficient production of the target protein can be achieved by utilizing the transcription activator-like effector nucleases (TALENs) and clustered regularly interspaced short palindromic repeats (CRISPR)/CRISPR-associated 9 (cas9).

In this work, in the developed functional silk, Lamp1 is not only presented on the surface but also contained inside the fiber. Therefore, not only the improvements in the Lamp1 expression in the transgenic *B. mori* is required but also the silk processing is important to increase its practicality. When it comes to processing, Sato et al. demonstrated improved functionality through the micronization silk expressing single chain variable fragment (scFv)-conjugated FibL [11]. Therefore, Lamp1 contained in the inside of the fiber can be effectively utilized by refining the fiber and increasing its surface area. One of the advantages of silk materials is that they can be processed in various ways [29,30]. In addition to the fine particles, studies have been reported proposing to change the shape of the dissolved silk into gels [12], films [31,32], and even sponges [14]. By using these techniques, it is possible to improve the surface of the fiber by making it uneven or hollow. Furthermore, in the silk material, because it is possible to introduce a hydrophilic group by blending or copolymerizing with a hydrophilic substance, therefore, it can be potentially processed into a material suitable for a RE recovery process. In addition, improvement of the peptide design is also important. In order to design a peptide suitable for the silk surface, there is a need for optimization of the linker length considering amino acid substitution with improved metal recognition and orientation on the silk surface. The current recovery rate of RE is quite low, i.e., approximately 10%, but based on the transgenic silkworm developed in this study, further improvements can be achieved.

Since the development of the first transgenic *B. mori* [15], various functional silks expressing useful fusion proteins have been reported [20,33,34]. The greatest advantage of using the transgenic silkworms is that the functional fibers and proteins can be prepared at low cost. In recent years, progress has been seen in the large-scale breeding of transgenic silkworms [16,18,35]. In this study, we showed for the first time the potential of a RE recovery by functional silk expressing the RE recognition peptide. Since this silk exhibits the ability to recognize RE even when cut with scissors, it can be used repeatedly. Our proposed simple approach has the potential to be used for a simple RE recovery from actual metal ion mixed solution such as seawater and industrial wastewater under mild conditions without using an organic solvent.

## 3. Materials and Methods

### 3.1. Plasmid Construction

The DNA sequences of Lamp1 fused with linker and His tag were synthesized by custom oligonucleotide synthesis service (Eurofins Genomics, Tokyo, Japan). Detailed sequence of DNA and amino acid are listed in the Supplementary Figure S2. Three kinds of DNA fragments were ligated into the *piggyBac* vector with restriction sites of *Bam*H I and *Hin*d III. The vector of *piggyBac*, pBac[3xP3-DsRed2afm], was used in this study as reported previously [11,36].

### 3.2. Generation of Transgenic Silkworms

Transgenic silkworms were generated as described elsewhere [15]. The transgene plasmid DNA and a helper plasmid vector pHA3PIG coding for *piggyBac* transposase, each dissolved in 5 mM KCl and 0.5 mM phosphate buffer (pH 7.0) at a concentration of 0.2 mg/mL, were mixed and injected into the fertilized eggs of the wild type strain, w1-pnd, at 4 to 10 h post oviposition. Hatched larvae (G0) were reared on an artificial diet (Nihon Nosan, Kanagawa, Japan) at 25 °C until they developed into moths and permitted to mate with each other. Using fluorescent microscopy (MZ16FA, Leica Microsystems, Wetzlar, Germany), G1 embryos were screened for transgenic individuals with *DsRed2* expression 6 to 7 days after oviposition. Transgenic silkworms were reared and sib-mated for at least three generations.

### 3.3. Purification of the Recombinant Protein

The cocoons obtained from transgenic silkworms were boiled for 20 min in 0.5% (w/v) Ivory soap solution and, then, rinsed several times with distilled water to remove the sericin. The silk fibroin was then dissolved in 9 M lithium bromide by incubating for 14 h at 37 °C and adjusted to 5% (w/v) solution. The solution was dialyzed with 20 mM Tris (pH 8.0), 5 M urea, 0.1 wt% 2-mercaptoethanol overnight, and the protein was purified using a column chromatography. Subsequently, protein purification was performed using HisTrap HP columns (GE healthcare, IL, Chicago); this purified protein was then concentrated using Vivaspin-turbo 4 centrifugal filter units, 10 kDa MW (Sartorius, Göttingen, Germany). The concentrations of the purified proteins were calculated from their respective absorbances at 280 nm using the molar extinction coefficients (Bio Spectrophotometer, Eppendorf, Hamburg, Germany).

### 3.4. SDS-PAGE and Western Blotting

SDS-PAGE was performed on 12.5% polyacrylamide gels (Kanto Chemical, Tokyo, Japan) according to the general protocol. After electrophoresis, polyacrylamide gels were stained with the gel-code blue stain reagent (Thermo Fisher Scientific, Waltham, MA, USA). The molecular weights of the proteins were estimated using standard protein markers, Precision Plus Protein^TM^ unstained protein standards (Bio-Rad, Hercules, CA). After electrophoresis, the proteins were transferred onto a polyvinylidene difluoride (PVDF) membrane using an iBlot system (Thermo Fisher Scientific) and blocked with 5% skim milk (Wako Pure Chemical Industries) in TBS buffer (50 mM Tris-HCl, pH 7.4, 150 mM NaCl) for 2 h. After washing with TBS buffer, the membranes were incubated with anti-Lamp (Scrum, Tokyo, Japan) or anti-His antibody (MBL, Nagoya, Japan), for 1 h. Super-Signal West Femto Maximum Sensitivity Substrate (Thermo Fisher Scientific) was used as a detection reagent.

### 3.5. RE Reaction

In the RE reaction using the purified protein, the solution of 50 µM of purified protein was added to 5 mM lanthanide nitrate (Ln(NO_3_)_3_) in 50 mM MES buffer (pH 6.0), and incubated for 0.5 to 24 h at 20 °C. In the RE reaction using the crushed silk, the silk crushed by an automatic milling apparatus was used. The silk cocoons obtained from each silkworm were chopped into pieces 5 to 10 mm in length, then, each sample was quenched with liquid nitrogen. The quenched sample was milled for 10 min by an auto mill mixer at 1200 rpm with stainless steel crusher (Automill TK-AM5, Tokken, Chiba, Japan). The 20 mg of this crushed silk was added to 5 mM lanthanide nitrate (Ln(NO_3_)_3_) in 50 mM MES buffer (pH 6.0) and incubated for 1 h at 20 °C. After the incubation, each sample was centrifuged at 15,000 rpm for 10 min, then, the collected supernatant solution was used for the analysis of RE concentration. The precipitate was washed thrice with 50 mM MES buffer (pH 6.0) and again twice with distilled water. After drying, each sample was used for SEM–EDX analysis.

### 3.6. SEM and EDX

The SEM-EDX analysis was performed using a TM3000 microscope (Hitachi high-technologies, Tokyo, Japan) operated at 15 keV. Each purified protein or crashed silk after the reaction was centrifuged at 15,000 rpm for 10 min. The supernatant was removed, and the precipitate was washed three times with pure water. The samples dispersed in water were dropped onto carbon tape or a nano-percolator (JEOL, Tokyo, Japan) and dried under atmospheric conditions before obtaining the images.

### 3.7. Ion-Exchange Chromatography

Ion-exchange chromatography to quantify the RE concentration was carried out using IonPac CG5A Guard column and CS5A analytical column (Thermo Scientific), under the following reaction conditions: For mobile phase A, 160 mM oxalic acid, 100 mM potassium hydroxide, and 200 mM tetramethylammonium hydroxide and for mobile phase B, 160 mM diglycolic acid and 190 mM potassium hydroxide. Detection reagent was 0.5 mM 4-(2-pyridylazo) resorcinol (PAR). Flow rate was 1.2 mL/min. Dy(NO_3_)_3_ was used as a standard solution for the calibration of a standard curve.

## 4. Conclusions

We demonstrated for the first time, to our knowledge, the potential of a transgenic fiber, made using functional silk expressing a RE-recognition peptide, to recover RE. This silk has a RE-recognizing ability and this function can be further improved by rough crushing. Moreover, the potential reusability of the silk particles has been illustrated. Although the current recovery rate of RE is low (~10%), based on the approach developed in this study, further improvements in the yields can be achieved. The great advantage of this material is that the RE can be easily and directly recovered without using an organic solvent or harmful conditions. Given its increasing utilization in the production of advanced material, the demand for the supply of RE is expected to increase. Hence, large scale cost- and energy-efficient technologies would be required. Our findings showing the RE recovery using functional silk has the potential to be an environmentally friendly technique for simple RE metal recovery.

## 5. Patents

Patent applications have been filed for the technology described in this publication.

## Supplementary Materials

The Supplementary Materials are available online. Figure S1: Genomic Southern blot analysis of transgenic silkworms, Figure S2: DNA and amino acid sequence of the Lamp1 protein.
