# Supplementary material for: Direct Recovery of the Rare Earth Elements Using a Silk Displaying a Metal-Recognizing Peptide
Source: Molecules. 2020 Feb 10;25(3):761. doi: 10.3390/molecules25030761 (PMC7037070; doi:10.3390/molecules25030761)
Supplement: Supplementary file 1 [file molecules-25-00761-s001.pdf]

Supplementary Materials

Direct Recovery of the Rare Earth Elements Using a Silk Displaying a Metal-Recognizing Peptide

Nobuhiro Ishida [1], Takaaki Hatanaka [1] , Yoichi Hosokawa [1] , Katsura Kojima [2] , Tetsuya Iizuka [3] , Hidetoshi Teramoto [2] , Hideki Sezutsu [3] and Tsunenori Kameda [2,*]

[1] Strategic Research Division, TOYOTA Central R&D Labs, Inc., 41-1, Yokomichi, Nagakute, Aichi 480-1192, Japan.
[2] Silk Materials Research Unit, Division of Biotechnology, Institute of Agrobiological Sciences, National Agriculture and Food Research Organization (NARO), 1-2, Owashi, Tsukuba, Ibaraki 305-8634, Japan.
[3] Transgenic Silkworm Research Unit, Division of Biotechnology, Institute of Agrobiological Sciences, National Agriculture and Food Research Organization (NARO), 1-2, Owashi, Tsukuba, Ibaraki, 305-8634, Japan.

*Address correspondence to: Silk Materials Research Unit, Division of Biotechnology, Institute of Agrobiological Sciences, National Agriculture and Food Research Organization (NARO), 1-2, Owashi, Tsukuba, Ibaraki 305-8634, Japan..
Dr. Tsunenori Kameda
E-mail: kamedat@affrc.go.jp

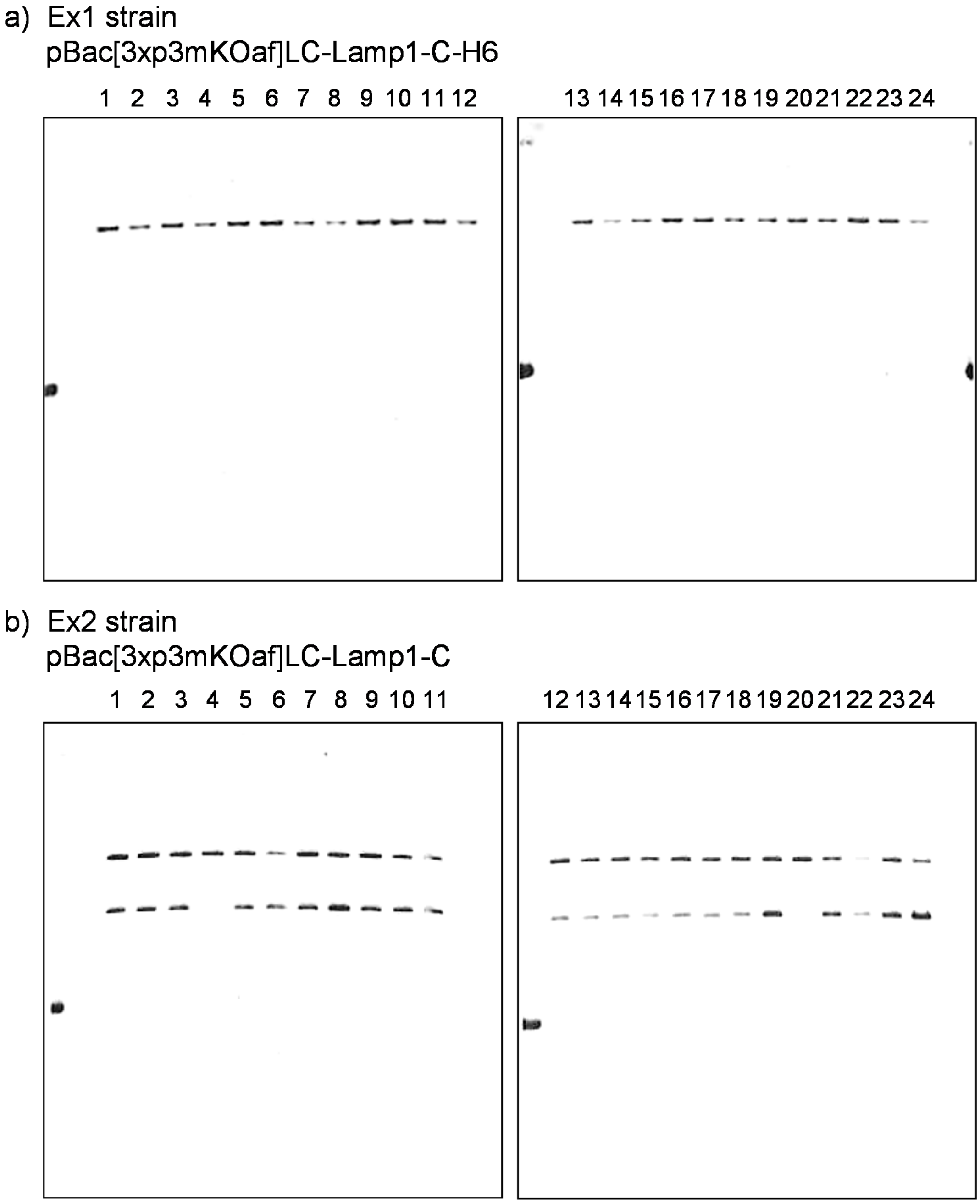

Supplementary Figure S1: Genomic Southern blot analysis of transgenic silkworms

The Probe DNA used here was the same as we reported previously (Kojima et al. (2007) A new method for the modification of fibroin heavy chain protein in the transgenic silkworm. *Biosci. Biotechnol. Biochem*. 71: 2943–2951).

a) Ex1 strain
pBac[3xp3mKOaf]LC-Lamp1-C-H6

```
aag ggt ggt gga tct ggc ggc ggt tct tgt tta tgg gga gac gta tca      48
Lys Gly Gly Gly Ser Gly Gly Gly Ser Cys Leu Trp Gly Asp Val Ser
1               5                   10                  15

gaa ctg gac ttc tta tgc tcc ggc ggc ggt tca cat cac cac cat cac      96
Glu Leu Asp Phe Leu Cys Ser Gly Gly Gly Ser His His His His His
            20                  25                  30

cac tga                                                             102
His
```

b) Ex2 strain
pBac[3xp3mKOaf]LC-Lamp1-C

```
aag gga ggc ggc tca ggg ggg ggt tcg tgt tta tgg gga gac gta tca      48
Lys Gly Gly Gly Ser Gly Gly Gly Ser Cys Leu Trp Gly Asp Val Ser
1               5                   10                  15

gag ctc gac ttc tta tgc tcg tag                                      72
Glu Leu Asp Phe Leu Cys Ser
            20
```

Supplementary Figure S2: DNA (upper) and amino acid (lower) sequence of the Lamp1 insert fragment

Sequence of insert fragment for vector construction. The yellow part indicates the linker and the green part indicates Lamp1.
